# Larotrectinib in NTRK Fusion-Positive High-Grade Glioneuronal Tumor: A Case Report

**DOI:** 10.7759/cureus.31449

**Published:** 2022-11-13

**Authors:** Ramya Tadipatri, Jennifer Eschbacher, Ekokobe Fonkem, John Kresl, Amir Azadi

**Affiliations:** 1 Neurology, Flagstaff Medical Center, Flagstaff, USA; 2 Pathology, Barrow Neurological Institute, Phoenix, USA; 3 Neuro-Oncology, Barrow Neurological Institute, Phoenix, USA; 4 Radiation Oncology, Radiosurgery, Phoenix CyberKnife and Radiation Oncology Center, Phoenix, USA; 5 Neuro-Oncology, Banner Health, Phoenix, USA

**Keywords:** glioma, larotrectinib, ntrk1, ntrk, glioneuronal

## Abstract

Glioneuronal tumors are rare central nervous system tumors with heterogeneous histological and molecular features. While the majority are low grade, a small percentage can behave aggressively. Due to the rarity of these tumors, there is no consensus on how to treat high-grade glioneuronal tumors, and they are often managed similarly to glial tumors. With the advent of molecular profiling, management decisions are increasingly determined by molecular alterations in the tumor rather than the tumor type, which can be a useful approach for tumor types that do not have robust supportive clinical trial data due to low prevalence. We present a case of an 18-year-old patient with a high-grade glioneuronal neoplasm initially treated with craniospinal irradiation, vincristine, and cyclophosphamide. He presented eight years later with a recurrent tumor and was found to be positive for MEF2D-NTRK1 fusion. He was treated with surgical resection and postoperative intensity-modulated radiation therapy (IMRT; 55.8 Gy) with concurrent temozolomide, followed by the NTRK inhibitor larotrectinib. He achieved a radiographic response, with a decrease in residual enhancement and radiographic improvement over the course of treatment. He remained in clinical and radiographic remission for six months. This demonstrates the successful treatment of a high-grade glioneuronal NTRK fusion-positive tumor with larotrectinib, which has only been previously reported once in the literature.

## Introduction

Glioneuronal tumors are rare central nervous system tumors consisting of glial and neuronal cells defined histologically and increasingly classified based on molecular features [1]. Under the 2021 WHO guidelines, glioneuronal and neuronal tumors are subclassified into ganglioglioma, desmoplastic infantile ganglioglioma/astrocytoma, dysembryoplastic neuroepithelial tumor, papillary glioneuronal tumor, rosette-forming glioneuronal tumor, myxoid glioneuronal tumor, diffuse leptomeningeal glioneuronal tumor, gangliocytoma, multinodular and vacuolating neuronal tumor, dysplastic cerebellar gangliocytoma (Lhermitte-Duclos disease), central neurocytoma, extraventricular neurocytoma, and cerebellar liponeurocytoma [2]. The most common histologic subtype is ganglioglioma, a WHO grade 1 tumor often characterized by mutations in the MAP kinase pathway, most notably the BRAF V600E mutation, KRAS mutation, NF1 mutation, or FGFR mutation or fusion [3]. While the majority of glioneuronal tumors are low-grade, a small percentage can have malignant histologic features and behave aggressively [4]. Here, we describe a case of a high-grade glioneuronal tumor found to harbor an NTRK fusion, which was successfully treated with larotrectinib. The relatively recent approval of larotrectinib combined with the rarity of glioneuronal tumors contributes to the uniqueness of this case.

## Case presentation

An 18-year-old, previously healthy male presented to an outside facility in 2013 with headaches and was found to have a 5.4 x 4.9 cm posterior fossa mass. He underwent resection, with pathology consistent with a high-grade glioneuronal neoplasm. The tissue was reviewed by multiple pathologists and was described as a ganglion cell tumor representing either medulloblastoma with extensive maturation or gangliocytoma with anaplastic features. He was treated with weekly vincristine and craniospinal radiation, with subsequent MRI showing improvement in the overall disease. He then received six cycles of consolidation treatment with vincristine and cyclophosphamide, with a 50% vincristine dose reduction due to peripheral neuropathy. Posttreatment MRI was stable with no evidence of active disease. He remained in remission for the next three years, with serial MRIs showing stable mild enhancement at the resection cavity, and he was subsequently lost to follow-up.

**Figure 1:**
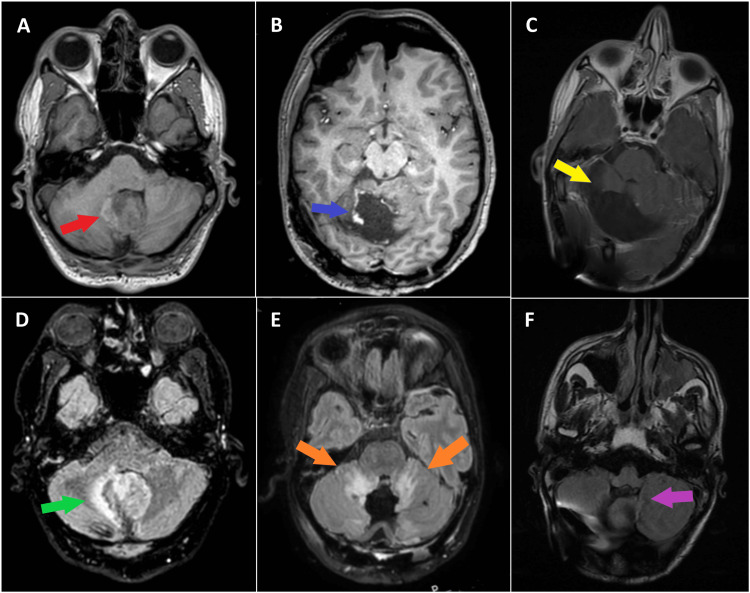
Serial MRIs of a Young Male With Recurrent High-Grade Glioneuronal Tumor Treated With Larotrectinib The top row depicts serial gadolinium-enhanced T1-weighted MRI images at recurrence with a posterior fossa mass with areas of enhancement (red arrow) (A), postoperatively with residual enhancement at the resection cavity (blue arrow) (B), and after six months of larotrectinib treatment with resolution of enhancement (yellow arrow) (C). The bottom row depicts serial, T2-weighted, fluid-attenuated inversion recovery (FLAIR) MRI images at recurrence with a T2 hyperintense mass and surrounding vasogenic edema (green arrow) (D), postoperatively with persistent vasogenic edema surrounding the resection cavity (orange arrows) (E), and after six months of larotrectinib treatment with resolution of the abnormal T2 signal (purple arrow) (F).

**Figure 2:**
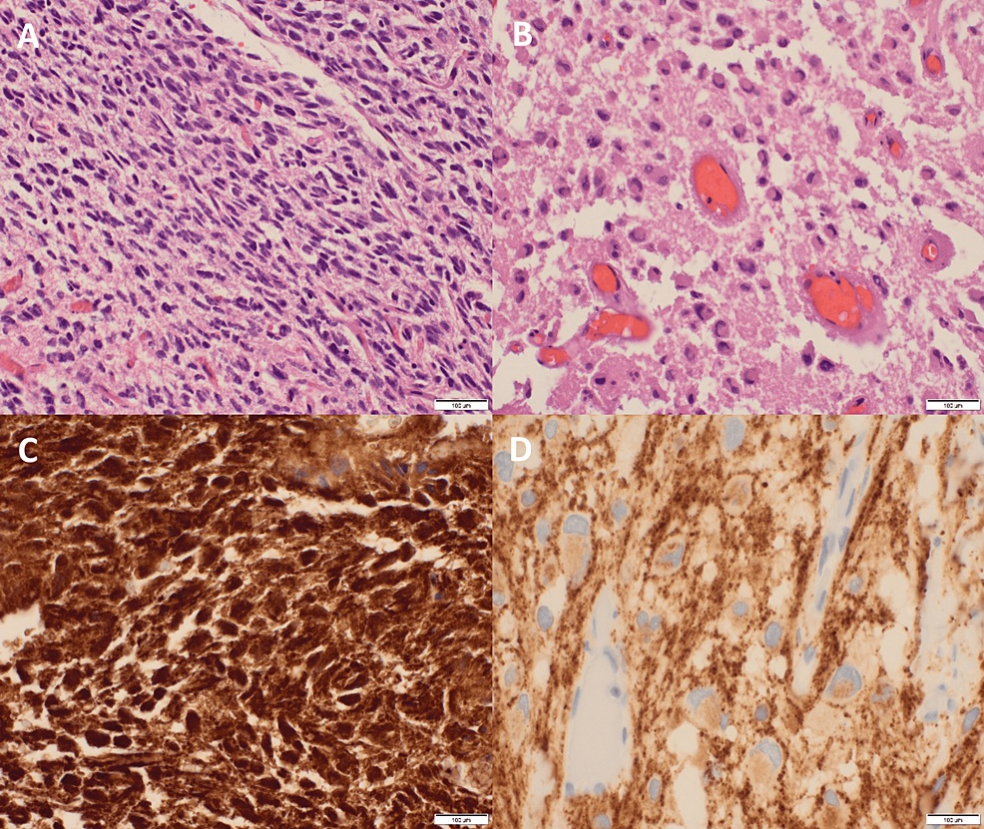
Tumor Histology of a Young Male With a Recurrent High-Grade Glioneuronal Tumor Hematoxylin and eosin-stained slides show a proliferation of atypical glia (200x) (A). Numerous atypical cells with neuronal features are seen (200x) (B). GFAP is positive in some of the atypical glia (400x) (C). Synaptophysin shows positivity in the neoplastic neuronal component (400s) (D).

In November 2020, he presented to our facility with three weeks of headache and imbalance and was found on CT to have an increase in the size of a 3.2 cm partially calcified mass in the surgical bed of the cerebellar vermis. MRI demonstrated an enhancing mass of the fourth ventricle with invasion into the superior cerebellum, with narrowing of the fourth ventricle but no evidence of hydrocephalus (Figure 1). He underwent near-total resection of the mass, pathology consistent with a high-grade glioneuronal tumor with malignant glial and neuronal components and a Mib-1 labeling index of 11% (Figure 2). Next-generation sequencing was positive for an NTRK1 mutation by copy number abnormality testing and demonstrated a tumor mutational burden of 1. Further staging with spine imaging postoperatively revealed diffuse subdural thickening and enhancement, which was favored to represent postoperative changes versus metastatic disease. A lumbar puncture was performed and demonstrated negative cytology for malignancy.

In January 2021, he received IMRT with 55.8 Gy in 31 fractions along with concurrent temozolomide. This was followed by one cycle of maintenance temozolomide. Follow-up confirmatory next-generation sequencing revealed MEF2D-NTRK1 fusion, NTRK1 amplification, and AKT3 amplification. There was no evidence of 1p or 19q deletion or KIAA1549-BRAF fusion. Methylation studies indicated no match to any methylation class in the current classifier. Based on these results, the final diagnosis was a glioneuronal tumor. Due to the discovery of MEF2D-NTRK1 fusion, temozolomide was discontinued, and he was started on larotrectinib in May 2021. He tolerated treatment with no reported serious adverse events. Surveillance MRI images showed decreased residual enhancement and radiographic improvement. He continues to be in remission with no evidence of radiographic recurrence or progression as of June 2022.

## Discussion

The NTRK genes NTRK1, NTRK2, and NTRK3 code for three transmembrane high-affinity tyrosine kinase receptors for nerve growth factors that play a role in central nervous system development. NTRK fusions lead to constitutively active receptors and have been identified in some types of cancer. While NTRK fusions are only observed in <1% of tumors overall, they can be pathognomonic for particular rare neoplasms, including breast secretory carcinomas, mammary analog secretory carcinoma of the salivary gland, infantile fibrosarcoma, and congenital/infantile mesoblastic nephroma [5].

Among pediatric central nervous system tumors, NTRK alterations have been observed in both low and high-grade gliomas. Among adults, NTRK alterations are most commonly observed in IDH wildtype glioblastoma, with NTRK fusions seen in 0.5-2.6% of glioblastomas. A wide variety of NTRK fusions have been described [5,6].

Larotrectinib was approved by the FDA in 2018 for NTRK fusion-positive solid tumors [6]. Among nine patients with NTRK fusion-positive primary central nervous system tumors (6 gliomas, 3 not otherwise specified) treated with larotrectinib, disease control was achieved in all (8 of 9) evaluable patients [7]. Entrectinib, which targets NTRK fusions while also simultaneously targeting ALK and ROS1 fusion proteins, was approved the following year. More recently, Doz et al. reported on 33 central nervous system tumors, including both high-grade and low-grade glioma, treated with larotrectinib [8]. Tumor shrinkage was observed in 82% of patients, and the objective response rate was 30%. 

Literature on NTRK fusion in glioneuronal tumors is limited by the rarity of the tumor (Table 1). Kurozumi et al. describe a case of a 13-year-old female with a high-grade glioneuronal tumor, methylation profile suggestive of diffuse leptomeningeal glioneuronal tumor and positive for ARHGEF2-NTRK1 fusion. The patient was treated with radiotherapy and temozolomide and remained in remission at nine months post-treatment [9]. Alvarez-Breckenridge et al. identified NTRK fusions (STRN3-NTRK2, WNK2-NTRK2, and BCAN-NTRK1) in three patients with low-grade glioneuronal tumors and reported the first case of successful treatment of a glioneuronal tumor with NTRK inhibition. The patient with BCAN-NTRK1 fusion was treated with entrectinib in a phase 1 study and demonstrated a 60% reduction in tumor size after nine months of therapy. Two months later, however, the patient had radiographic progression, and treatment was discontinued [10]. Boyer et al. describe a case of a 53-year-old male with a high-grade glioneuronal tumor positive for STRN1-NTRK2 fusion. The patient was treated with radiotherapy and temozolomide and had progression of disease a month after completing treatment. The patient then was treated with larotrectinib and remained in remission at 11 months posttreatment [11]. The NAVIGATE and SCOUT trials included six patients with rare central nervous system tumors harboring an NTRK mutation and treated with larotrectinib, including glioneuronal tumors among others, although a further breakdown or information regarding tumor grade was not provided. Among these patients, one demonstrated a complete response, one demonstrated a partial response, and the remainder had stable disease [8].

**Table 1:** Cases of NTRK Fusion-Positive Glioneuronal Tumors Reported in Literature RT: radiotherapy; TMZ: temozolomide

Source	Fusion Partner	Tumor Grade	Recurrent vs Newly Diagnosed	Agent Used	Outcome
Kurozumi et al. 2019	ARHGEF2	High	Newly diagnosed	RT/TMZ	Remission at 9 months
Alvarez-Breckenridge et al. 2017	STRN3	Low	Not reported	Not reported	Not reported
Alvarez-Breckenridge et al. 2017	WNK2	Low	Not reported	Not reported	Not reported
Alvarez-Breckenridge et al. 2017	BCAN	Low	Newly diagnosed	Entrectinib	60% tumor reduction at 9 months, then progression at 11 months
Boyer et al. 2021	STRN1	High	Recurrent	Larotrectinib	Remission at 11 months
Current case	MEF2D	High	Recurrent	Larotrectinib	Remission at 6 months

Our patient did receive radiotherapy and temozolomide prior to the initiation of larotrectinib. However, high-grade glioneuronal tumors historically have a median overall survival of 24.7 months and median progression-free survival of 8.0 months, with minimal benefit achieved through radiotherapy and temozolomide [12,13]. Furthermore, our patient presented with recurrent disease, which tends to be more refractory to treatment, and only received one cycle of maintenance temozolomide, rather than six cycles per standard of care. The observed survival benefit can therefore most likely be attributed to larotrectinib.

## Conclusions

The management of rare tumors poses a challenge due to the paucity of evidence to support treatment decisions. While NTRK inhibition has been described in gliomas, data on glioneuronal tumors are limited. As next-generation testing becomes increasingly available, rare tumors can be managed based on targetable molecular markers. Our case illustrates the successful treatment of a high-grade glioneuronal tumor with NTRK inhibition, which has only been previously reported once in the literature.
